# Weekly and Holiday-Related Patterns of Panic Attacks in Panic Disorder: A Population-Based Study

**DOI:** 10.1371/journal.pone.0100913

**Published:** 2014-07-09

**Authors:** Li-Ting Kao, Sudha Xirasagar, Kuo-Hsuan Chung, Herng-Ching Lin, Shih-Ping Liu, Shiu-Dong Chung

**Affiliations:** 1 Graduate Institute of Life Science, National Defense Medical Center, Taipei, Taiwan; 2 Sleep Research Center, Taipei Medical University Hospital, Taipei, Taiwan; 3 Arnold School of Public Health, Department of Health Services Policy and Management, University of South Carolina, Columbia, South Carolina, United States of America; 4 Taipei Medical University Hospital, Department of Psychiatry, Taipei, Taiwan; 5 Taipei Medical University, School of Medicine, Department of Psychiatry, Taipei, Taiwan; 6 Taipei Medical University, School of Health Care Administration, Taipei, Taiwan; 7 Department of Urology, National Taiwan University Hospital and College of Medicine, Taipei, Taiwan; 8 Division of Urology, Department of Surgery, Far Eastern Memorial Hospital, Ban Ciao, Taipei, Taiwan; 9 School of Medicine, Fu-Jen Catholic University, New Taipei City, Taiwan; Institute of Psychiatry, United Kingdom

## Abstract

**Background:**

While chronobiological studies have reported seasonal variation in panic attacks (PA) episodes, information on the timing of PA by week-days may enable better understanding of the triggers of PA episodes and thereby provide pointers for suitable interventional approaches to minimize PA attacks. This study investigated weekly variation in potential PA admissions including associations with holidays using a population-based longitudinal, administrative claims-based dataset in an Asian population.

**Methods:**

This study used ambulatory care data from the “Longitudinal Health Insurance Database 2000. We identified 993 patients with panic disorder (PD), and they had 4228 emergency room (ER) admissions for potential PA in a 3-year period between 1 January 2009 and 31 December 2011. One-way analysis of variance (ANOVA) was used to examine associations between the potential PA admissions and holidays/weekend days/work-days of the week.

**Results:**

The daily mean number of potential PA admissions was 3.96 (standard deviation 2.05). One-way ANOVA showed significant differences in potential PA admissions by holiday and day of the week (*p*<0.001). Daily frequencies showed a trough on Wednesday-Thursday, followed by a sharp increase on Saturday and a peak on Sunday. Potential PA admissions were higher than the daily mean for the sample patients by 29.4% and 22.1%, respectively on Sundays and holidays. Furthermore, the weekly variations were similar for females and males, although females always had higher potential PA admissions on both weekdays and holidays than the males.

**Conclusions:**

We found that potential PA admissions among persons with PD varied systematically by day of the week, with a significant peak on weekends and holidays.

## Introduction

Panic disorder (PD) is a widely prevalent severe anxiety disorders characterized by recurrent and unexpected panic attacks (PA) [1]. PA is generally characterized by intense fear or discomfort with least four somatic or cognitive symptoms, sometimes accompanied by agoraphobia [2], [3]. In the United States, an estimated that 4.8% of the adult population is affected by PD over the lifetime with or without agoraphobia [4]. Studies show that PD is costly for patients and society due to increased use of medical resources and reduced work productivity [5]–[7]. Studies of temporal variations in PA episodes may provide pointers to the underlying etiological factors and may enable intervention approaches to minimize the severity and frequency of PA.

The risk factors for PA remain unclear [5] despite studies suggesting possible associations with lifestyle [8], [9], psychosocial [5], [10], and environmental factors [8], [10], [11]. Chronobiological studies have reported seasonal variations in PA episodes [10]–[12]. Studies from Australia and Japan indicate higher incidence in summer and winter [10], [11]. However, very few studies on detailed temporal variation synchronous with the diurnal and weekly rhythms of people's lives have been documented. A study from Finland reported that the diurnal and weekly rhythms of people's lives are closely related to work and leisure times [13]. A finding of associations between PA onset and the day of the week may provide practical guidance on the context and factors that trigger a PA and thus provide pointers for research and intervention programs. This study investigated variations in potential PA admissions among persons with a PD diagnosis by day of the week using a population-based dataset in an Asian population.

## Methods

### Data source

This study used ambulatory care data from the “Longitudinal Health Insurance Database 2000 (LHID2000)” published by the National Health Research Institute (NHRI) of Taiwan. The LHID2000 consists of de-identified cumulative claims data on one million randomly selected enrollees of Taiwan's National Health Insurance system designed to be representative of the total enrollee population as of December 2000. The LHID2000, which was open to the researchers in Taiwan, was available from the NHRI (http://nhird.nhri.org.tw/date_01.html). This study is based on de-identified secondary data from the LHID2000 released by the NHRI without restrictions for research purposes. As such it was exempted from full review by the Taipei Medical University's Internal Review Board (IRB) and is in compliance with the international ethical standards.

### Study Sample

We identified all patients with a principal diagnosis of PD (ICD-9-CM code 300.01) in the LHID2000 with an ambulatory psychiatric care treatment claim between January 2008 and December 2008 (n = 1,716). To ensure validity of diagnosis of study patients we included patients who had at least two PD diagnoses in ambulatory care claims of which at least one was made by a certified psychiatrist. We restricted our study to only those who had ever been admitted into an emergency room (ER) for potential PA between 1 January 2009 and 31 December 2011 for a study sample of 993 patients with PD.

We defined potential PA if a PD patient admitted to an ER was diagnosed with a PA-relevant symptom such as dizziness/giddiness, headache, hyperventilation, chest pain, palpitations, abdominal pain, and anxiety state without a definitive diagnosis of a related physical condition. We used symptoms identified in the Diagnostic and Statistical Manual of Mental Disorders, fourth edition (DSM-IV) criteria for PA [3], [14]. Taiwan has a well-developed and well-distributed network of emergency medical services which is covered by NHI, which in turn provides medical care coverage to 99% of Taiwan's citizens with a common benefit package. Therefore, potential PA admissions likely reflect the population-based incidence of PA in Taiwan. Readmission to the ER within seven days of the first visit was not counted, being treated as part of the same episode. A total of 4,228 potential PA admissions were identified.

### Statistical analysis

Statistical analyses were performed with the Statistical Package for the Social Sciences (SPSS 10.0 for Windows, 1997, SPSS, Chicago, IL). Potential PA admissions were categorized by week-day of visit (seven days) and an additional variable of holiday during week-days. Holidays were defined as national holidays in Taiwan. We present daily variations within the week expressed as percentages of the total and for each gender group. One-way analysis of variance (ANOVA) was used to examine associations between the daily frequency of potential PA admissions and holidays/weekend days/work-days of the week. Percentage of variation from the sample daily mean were calculated, the difference between each weekday's potential PA admissions and the sample daily mean, divided by the latter. A two-sided p value of ≤0.05 was considered statistically significant.

## Results

**Figure 1:**
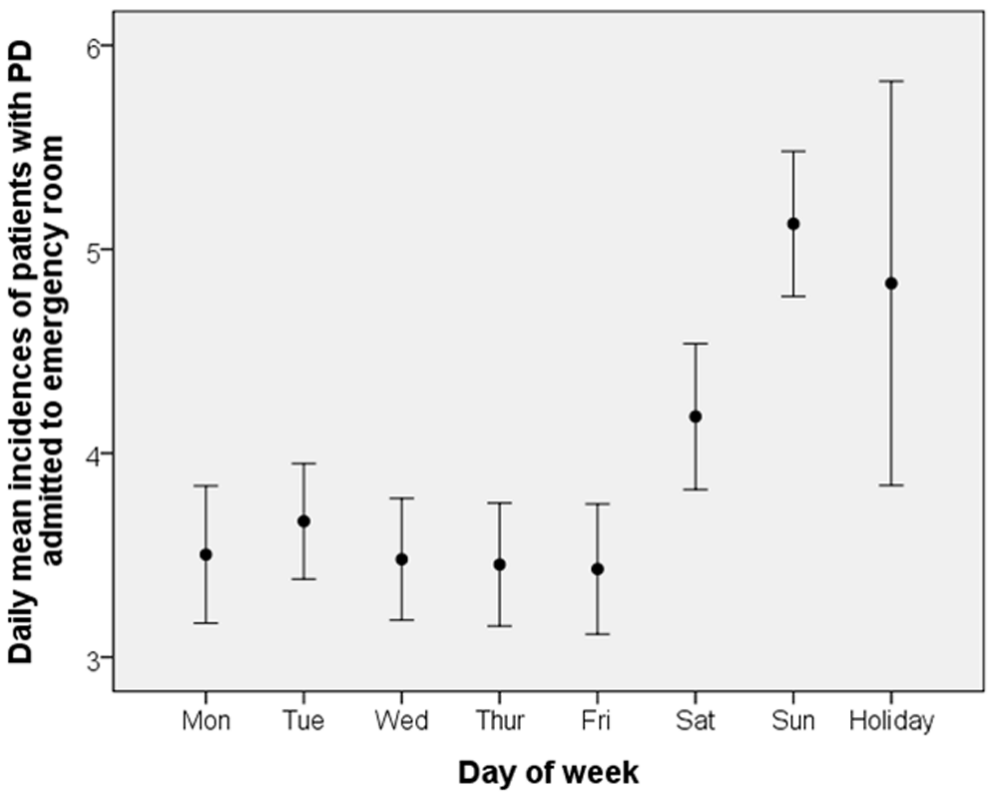
Daily mean incidences of patients with PD admitted to emergency departments and 95% confidence interval in Taiwan.

The distribution of sample patients by demographic characteristics is presented in Table 1. The majority of patients were female (62.7%), and the mean sample age was 48.4 years (standard deviation 14.0 years). Almost half of the sample patients resided in northern Taiwan which has Taipei the most populated city and region in Taiwan. Of total 4,228 potential PA admissions during 2009–2011, 1,519, 1,391, and 1,318 took place in 2009, 2010, and 2011, respectively. The mean daily number of potential PA admissions was 3.96 (standard deviation 2.05). Table 2 and Figure 1 present the daily mean number of potential PA admissions by week days. Sundays and holidays had higher mean potential PA admissions than other week days/non-holiday week days. Consistent with this finding, one-way ANOVA showed significant differences by holiday and by the day of the week (*p*<0.001), with a potential PA admission more likely to occur on a Sunday and on holidays than on other days within each gender group.

**Table 1:** Demographic characteristics of patients identified in 2008 with a panic disorder diagnosis and admitted to emergency departments in Taiwan (n = 993).

Variable	n (%)
Age (years)	
<25	71 (7.2)
25–34	113 (11.4)
35–44	228 (23.0)
45–54	223 (22.5)
55–64	142 (14.3)
≧65	216 (21.8)
Sex	
Male	371 (37.4)
Female	622 (62.7)
Geographic region	
Northern	482 (48.5)
Central	246 (24.8)
Southern	226 (22.8)
Eastern	39 (3.9)
Urbanization level	
1 (Most urbanized)	286 (28.8)
2	295 (29.7)
3	140 (14.1)
4	125 (12.6)
5 (Least urbanized)	147 (14.8)
Monthly income	
NT$1-15,840	397 (40.0)
NT$15,841-25,000	350 (35.3)
≥NT$25,001	246 (24.8)

**Table 2:** Mean daily ER admissions 2009–11 for PA pooled by day of the week and holidays in Taiwan.

Variable	Mean	SD	Days	Minimum	Maximum
Monday	3.50	2.08	149	0	10
Tuesday	3.67	1.77	153	0	8
Wednesday	3.48	1.86	152	0	8
Thursdays	3.46	1.89	154	0	9
Friday	3.43	2.01	155	0	9
Saturday	4.18	2.21	150	0	12
Sunday	5.13	2.22	152	1	12
Holiday	4.83	2.65	30	0	11

**Figure 2:**
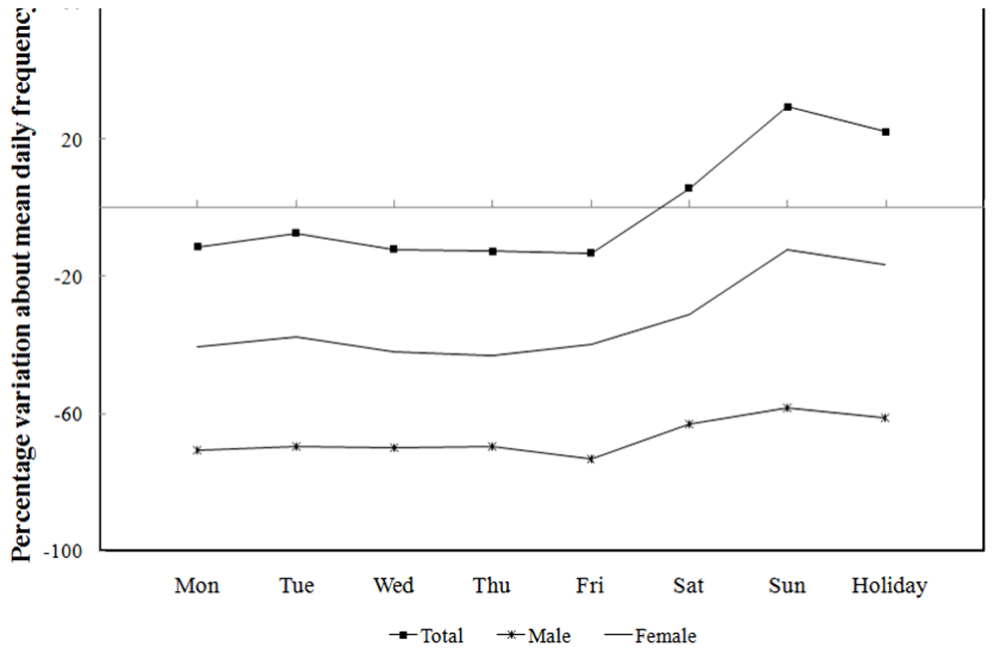
Percent variation of mean daily average of PA ER admissions relative to sample mean, total sample and by gender (2009–2011).

The percent variations of daily potential PA admissions from the total sample mean of each gender are shown in Figure 2. The weekly variations were similar for females and males, although females always had higher potential PA admissions on both weekdays and holidays than the males. The pattern showed a trough on Wednesday-Thursday, followed by a sharp increase on Saturday and a peak on Sunday. Sunday and holidays had 29.4% and 22.1% higher potential PA admissions, respectively, than the daily mean for the total sample.

## Discussion

This population-based study found a systematic variation with the day of the week for potential PA admissions among PD patients, with higher admission rates on Sundays and holidays. The Sunday peak is preceded by a spike on Saturdays, which in turn is preceded by a trough in the middle of the work-week.

The study's strengths in the present study include the use of a nationwide population-based dataset with a single-payer system and generous health benefit coverage, one of the few such contexts and datasets available in the world. These features mitigate selection bias. Because it uses provider-based data with diagnostic codes and care information the study does not suffer from recall bias, a frequent weakness of studies that rely on patient recall.

There are some study limitations including the lack of information on patients' use of tobacco, alcohol, emotional stress and working conditions that may mediate some of our findings. Second, PD patients admitted to the ER may have had other medical conditions that share symptoms with PA. Mitigating this issue is our ability to screen these patients for other admission diagnoses before attributing their potential PA admissions, and our use of symptoms identified in the Diagnostic and Statistical Manual of Mental Disorders, fourth edition (DSM-IV) criteria for PA [3], [14].

Third, ERs may be the only (or predominant) medical care source available on weekends and holidays which could underlie our finding of higher visit rates on these days. Thus mild cases reporting on weekends that would be treated in outpatient clinics on week-days may be contributing to the excess events observed on weekends and holidays. To mitigate this issue, we considered only ER admissions, not outpatient visits. The patient should have a significantly serious symptom level to merit an ER admission for observation. ER admission for observation is provided on all days of the week. With long wait times that are typical of outpatient providers in Taiwan, and prompt care typical of ERs, it would be atypical for PA patients with significant symptoms to visit an outpatient care setting on a weekday. Additionally the observed gradual decrease on Mondays and the mid-week trough may support that our findings are robust to care-seeking behavior differences by day of the week.

Finally, some PD patients with mild PA symptoms would not be admitted to ER and would not be recorded in the database. Therefore, the results in our study might be not generalized to the PD population as a whole in Taiwan.

Many studies have investigated diurnal, weekly or seasonal variations in the onset and mortality of various diseases, acute myocardial infarction onset [15], [16], stroke onset [17], [18], cardiac arrest incidence [19] and sudden cardiac death [20] etc. There are few studies of PA variation, being limited to two studies of seasonal variation showing increased PA onset among PD patients in summer and winter [10], [11]. The authors concluded that PD patients may be more sensitive to seasonal changes than the general population with the summer increase attributed to meteorological factors, and the winter increase to meteorological or sociocultural factors including busy work schedules and year-end parties) [10].

One study in Spain explored ER visit rates for PA on weekdays and weekends. Contrary to our findings, they found that no difference in the mean number of PA emergency room visit rates between weekdays and weekends among PD patients [12]. The difference between the two study findings may be due to two reasons The Spanish study used data from selected hospitals and therefore may reflect hospital policy biases. Second, their study presented comparisons of mean pooled frequencies of all ‘weekdays’ and both ‘weekend days’, which does not permit distinctions between the midweek trough and the tapering increases and declines preceding and succeeding the mid-week trough. Pooling daily frequencies would bias results towards the null hypothesis. A study by Tuomisto et al. studied the diurnal and weekly rhythms of certain relevant physiological and psychological variables [13].

Our results showed that PD patients have higher potential PA admissions on weekends and holidays than on weekdays regardless of gender. One of the suspected reasons might be the effects of people's life rhythms related to work and leisure. In particular, our findings may resonate with several studies that explored the association of leisure and occupation with mental health conditions. Adverse cross-sectional associations of increased leisure time with spurts in mood disorders and depression, particularly the time duration spent on passive or inactive time-use behaviors (such as television-watching and casual computer use). These associations are documented among vulnerable subgroups such as adolescents and epileptics [21], [22]. The authors noted the need for temporal/longitudinal studies to investigate causality of the observed associations. Other authors have noted the beneficial effects of occupational therapy on psychological distress, dysfunctional symptoms and outcomes among schizophrenia patients [23], [24], and among community-dwelling adults with a mental health diagnosis [25]. It is possible that routine engagement in work during the work week may explain reduced potential PA admissions among PD patients.

Another potential explanation for our findings is that these persons may experience heightened anticipation of the approaching work stress on weekends and holidays. Melchior et al. reported that work stress may precipitate depression and anxiety in healthy young workers [26]. Other studies show that acute stress may trigger PA in predisposed persons with high anxiety levels such as PD patients [27]–[29]. It does not appear to be well supported by the pattern of our findings because in such case, Saturdays should be associated with a significant dip following the week of work stress, and followed by a rise on Sundays. Collectively, the literature on work and leisure in the context of mental health is consistent with our finding of weekly and holiday-related variations in potential PA admissions.

Another explanation for our findings may be related to alcohol and illicit drug consumption facilitated on weekends and holidays. Studies show increased alcohol and drug abuse propensity brought about by panic, which itself could be induced by withdrawal stress among long-time alcohol or drug users [30], [31]. A large European study revealed that significantly higher adjusted daily ethanol intake on weekends compared to the weekdays in many countries [32]. Increased abuse of drugs such as cocaine and ecstasy on weekends is documented in Milan [33]. Therefore, alcohol and illicit drug consumption could be the reasons behind our findings as they were not measured or controlled for and cannot be ruled-out.

In conclusion, the day-to-day fluctuation over the week in potential PA admissions among PD patients is significant and suggests the need for clinical research to identify the causes and design appropriate intervention to improve the quality of life of PD patients and to reduce costs associated with ER use.
